# Reply to Çiftci, S.; Aydin, B.K. Comment on “Lee et al. Accuracy of New Deep Learning Model-Based Segmentation and Key-Point Multi-Detection Method for Ultrasonographic Developmental Dysplasia of the Hip (DDH) Screening. *Diagnostics* 2021, *11*, 1174”

**DOI:** 10.3390/diagnostics12071739

**Published:** 2022-07-18

**Authors:** Si-Wook Lee, Hee-Uk Ye, Kyung-Jae Lee, Woo-Young Jang, Jong-Ha Lee, Seok-Min Hwang, Yu-Ran Heo

**Affiliations:** 1 Department of Orthopedic Surgery, Dongsan Medical Center, School of Medicine, Keimyung University, Daegu 42601, Korea; yhu4032@gmail.com (H.-U.Y.); oslee@dsmc.or.kr (K.-J.L.); 2 Department of Orthopedic Surgery, Korea University Anam Hospital, 73, Goryeodae-ro, Seongbuk-gu, Seoul 02841, Korea; opmanse@gmail.com; 3 Department of Biomedical Engineering, Keimyung University, Daegu 42601, Korea; segeberg@gmail.com (J.-H.L.); meen2378@naver.com (S.-M.H.); 4 Department of Anatomy, Dongsan Medical Center, School of Medicine, Keimyung University, Daegu 42601, Korea; chzh5240@naver.com

We thank Dr. Sadettin Ciftci for his comment on the key point issues in measuring the alpha and beta angle with Graf method. We appreciated his feedback. Specifically, Dr. Sadettin Ciftci pointed out that image and B angle measurements in our article are insufficient in order to be able to consider reliability of the AI method in the use of DDH screening [1].

Although the original Graf method describes the identification of 8 anatomical keys, it is used actually 3 landmarks with ultrasound [2]. The landmark is described that (1) the lower limb for DDH screening through alpha angle and beta angle measurement of os ilium; (2) the middle of the bony roof; (3) the labrum. In this study, the key points for measuring the alpha beta angle were set to 4, and the inclination of the parallel line of the iliac bone was limited to within 5 degrees. It is configured to evaluate quality [3]. 

In the case of beta angle, as the reader pointed out, it is already well known that the degree of agreement to the key point of the cartilaginous labrum is quite poor. And even as shown in the study of Simon et al, [4] in the comparison of beta-angle orthopedic surgeons and pediatricians, ICC was 0.34, in keeping with poor measurement reproducibility. This is probably the fundamental problem of ultrasound caused by the lack of clear boundaries in soft tissue labrum, and in our study, the ICC of beta angle for beta angle measurement using artificial intelligence rose to about 0.74. Although the use of alpha angle is preferred for DDH screening using ultrasound, it is thought that beta angle can also be used [1].
